# A Randomized, Double-Blind, Placebo-Controlled Study to Evaluate the Effect of *Limosilactobacillus fermentum* K8-Lb1 Postbiotic on Weight Management and Metabolic Health Outcomes

**DOI:** 10.3390/nu18081174

**Published:** 2026-04-08

**Authors:** Ekaterina Papazova, Susanne Mitschke, Christiane Laue, Jürgen Schrezenmeir

**Affiliations:** 1 Clinical Research Center Kiel, Kiel Center of Innovation and Technology, Schauenburgerstraße 116, D-24118 Kiel, Germany; 2 Citruslabs, Las Vegas, NV 89118, USA; 3 University Medicine, Johannes-Gutenberg University, D-55131 Mainz, Germany

**Keywords:** postbiotics, *L. fermentum*, weight management, body fat mass, metabolic syndrome

## Abstract

Background: Recent research has highlighted the potential of postbiotics for addressing obesity and associated metabolic disorders. In this randomized, double-blind clinical trial, the efficacy of a postbiotic product in managing overweight and associated parameters was assessed. Methods: Sixty individuals were randomized into two groups: one group (*n* = 30) received the Postbiotic (heat-killed *L. fermentum* strain K8-Lb1) and the other (*n* = 30) a Placebo control. Body weight, waist circumference, body composition, vital signs, blood biomarkers and questionnaires for quality of life, eating behavior, eating control and gastrointestinal symptoms were assessed. Results: After a 12-week intervention, body fat mass (primary parameter) was significantly (*p* = 0.016) reduced in the Postbiotic group (98.15 ± 3.32% of baseline) compared to the Placebo group (100.41 ± 3.39%). In line with this, body weight (*p* = 0.047) and waist circumference (*p* = 0.034) were significantly reduced and visceral fat tended to be reduced (*p* = 0.053). Accordingly, the Postbiotic group tended (*p* = 0.066) to feel more in control of their body weight. Despite weight loss, muscle mass tended (*p* = 0.062) to increase. ALT, AST and GGT tended to be reduced, which may indicate an improvement in liver steatosis. Estimated average glucose (eAG) differed significantly between the groups in individuals with normal fasting glucose levels. The ability to concentrate significantly (*p* = 0.014) improved. Conclusions: Under an ad libitum diet, the postbiotic *L. fermentum* strain K8-Lb1 reduced body fat mass, body weight, and waist circumference, improved the ability to concentrate, and showed a trend towards an increase in muscle mass. The results of this pilot trial need confirmation by a pivotal trial.

## 1. Introduction

Obesity is a significant public health concern with increasing prevalence. In the United States, a rate of 40.3% in adults was reported for the period from August 2021 to August 2023 [1]. The economic burden of obesity, using total deaths for estimation, was $3411.55 billion in 2024 [2], and the burden, defined as annual medical cost, was valued at nearly $173 billion in 2019 in the United States alone, where medical costs for adults with obesity are $1861 higher than for those with healthy weight [3].

Despite conventional recommendations for combating obesity, including healthy eating practices and regular exercise, the prevalence of severe obesity has almost doubled over the past two decades [4]. This escalating trend underscores the need for novel approaches to weight management.

Emerging evidence suggests a pivotal role of the gut microbiota in obesity, with alterations in the gut’s microbial profile linked to increased energy harvest and metabolic pathways contributing to obesity [5,6]. Targeting the gut microbiota through pro- and postbiotic interventions offers a promising strategy. Probiotics, live microorganisms with health benefits, and postbiotics, inanimate microbial components, have shown the potential to modulate the gut microbiota, enhancing gut integrity, counteracting inflammatory signaling, modulating anorexigenic and satiety signaling and counteracting the microbial shifts associated with obesity and related disorders [7,8,9,10].

Postbiotics present a novel avenue for addressing obesity and metabolic health outcomes. As products of probiotic bacteria, postbiotics offer the advantages of stability and safety, eliminating risks associated with live bacterial administration. They have also been implicated in improving metabolic and inflammatory markers, restoring gut microbe diversity, and providing overall beneficial effects in obesity management [11]. This approach aligns with the increasing recognition of the gut microbiome’s critical role in metabolic health, providing a novel pathway to combat obesity and its related complications.

The SlimBiotics postbiotic formula is the world’s first plant-based postbiotic targeting healthy weight management. The SlimBiotics postbiotic formula is derived from a probiotic *Lactobacillus fermentum* (updated taxonomy *Limosilactobacillus fermentum*) strain (K8-Lb1) isolated, among others, from Kimere, a spontaneously fermented pearl millet dough common among the Mbeere community of Kenya, East Africa [12].

In contrast to other *L. fermentum* strains from the same origin, which enhanced Th1 and Th2 responses, this strain suppressed T-helper cell 1 (Th1) and T-helper cell 2 (Th2) responses in human peripheral blood mononuclear cells (PBMCs) and, hence, showed anti-inflammatory properties [13]. K8-Lb1 was shown to differ from the Th1 response-enhancing *L. fermentum* strain K1 in various enzymes involved in the synthesis of glycans. A difference in the hydrophobicity properties of the surfaces of both strains indicated that this has an impact on their surfaces [13]. Proteoglycans at the surface of bacteria were shown to act as ligands for receptors, like toll-like receptors (TLRs) [14] and peptidoglycan recognition proteins (PGlyRPs) [15,16], which modulate the immune response. The specific surface properties of K8-Lb1, hence, explain why anti-inflammatory effects can be exerted by heat-killed K8-Lb1, even though anti-inflammatory metabolites, such as short-chain fatty acids (SCFAs), are not produced by these inanimate cells. Low-grade inflammation and LPS lead to attenuated CCK-induced satiation and dysregulation of anorexigenic and orexigenic hormones expressed in vagal afferent neurons, concomitant with hyperphagia and obesity development [9]. Fragments of proteoglycans, so-called muramyl dipeptides, were shown to stimulate GLP-1 and to improve insulin resistance, metabolic tissue inflammation and glucose tolerance [17]. *L. fermentum* strains contain the chaperone ClpL, which seems to operate through mechanisms similar to ClpB [18], which mimics α-MSH, a molecule involved in controlling appetite, thereby helping to reduce food intake and induce weight loss [19,20]. Indeed, a probiotic mixture of three anti-inflammatory L. *fermentum* strains of the same origin, including K8-Lb1, reduced body fat mass (BFM), body weight, body mass index (BMI), waist circumference, waist-to-height ratio, visceral adipose tissue and liver steatosis grade compared with the placebo in a randomized, double-blind clinical trial in individuals with abdominal overweight [21]. In an obesity model of *C. elegans*, this strain, K8-Lb1, suppressed fat accumulation in live and inanimate forms (unpublished data). Based on these findings, this first-in-man pilot study aimed to examine the effect of the postbiotic, heat-inactivated *L. fermentum* K8-Lb1 strain in reducing body fat mass (primary parameter), body weight and related parameters, including metabolic health outcomes, under an ad libitum diet.

## 2. Materials and Methods

### 2.1. Ethics

The protocol, informed consent forms, recruitment materials, and all participant materials were submitted to ARGUS Independent Review Board, 6668 S. Hidden Flower Way, Tucson, AZ 85756, USA (IRB), for review and approval. The protocol and consent form were approved before enrolling any participant. Ethical evaluation resulted in a written, stated favorable opinion on 10 April 2023.

The study was registered at ClinicalTrials.gov, trial registration number NCT05912699, on 22 June 2023.

The study was conducted in line with the principles of the Declaration of Helsinki, in accordance with the guidelines for Good Clinical Practice (ICH-GCP), with CONSORT 2025, and in accordance with the study protocol (study protocol No. 20277, version 2.0, dated 27 January 2023). Written informed consent was obtained from the study participants prior to any study-specific process.

The individual de-identified participant data (including the data dictionary), statistical code and any other materials can be accessed upon request from Citruslabs.

### 2.2. Study Design and Conduct

This study followed a double-blind, randomized, placebo-controlled design with two parallel arms following the principles of a superiority framework. The sponsor of the study made a decision on the sample size on a budgetary basis but had no role in further aspects of the design of the trial, in data analysis, interpretation of the results and manuscript preparation. Participants were randomly assigned to either the Postbiotic group or the Placebo (control) group in equal proportions and blinded to which group they were allocated. The random allocation sequence was generated by the study coordinator using Excel’s random number function. Participants were assigned sequentially as they enrolled. The study coordinator maintained a pre-generated randomization list in Excel and assigned participants to the next available allocation upon enrollment. The study team was blinded to treatment identity throughout the trial; products were labeled only as Product A or Product B, with the sponsor retaining the unblinding key. The study coordinator enrolled participants, assigned them to interventions and had access to the full randomization sequence. Allocation concealment was maintained through product blinding: products were identically labeled as Product A and Product B, with treatment identity known only to the sponsor. All study personnel were blinded to treatment allocation, including participants, study staff, and data analysts. Only the sponsor held the unblinding key. Data analysis was initially conducted blinded, with an unblinded analysis performed subsequently.

The intervention period lasted 12 weeks each.

The trial was conducted under a single coordinating organization. The places of recruitment and assessment were decentralized, with participants recruited remotely across multiple geographic locations and without a physical study site. The study was executed between 11 August 2023 (First Patient, First Visit) and 25 April 2024 (Last Patient, Last Visit).

**Figure 1:**
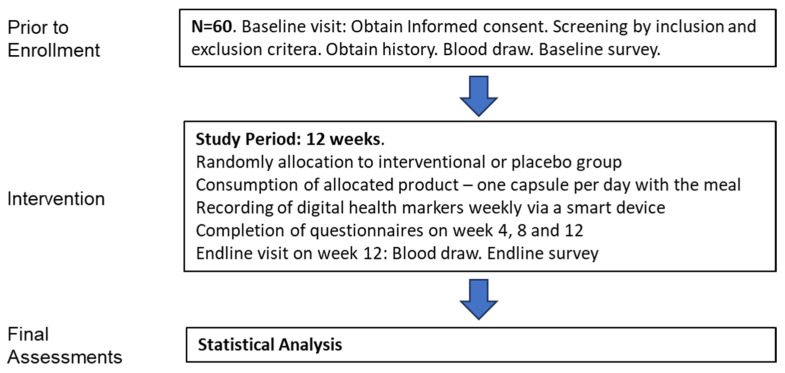
Study design.

Sixty female and male overweight individuals (BMI 25 to 32), aged ≥ 18 years, complying with the inclusion and exclusion criteria listed below, were enrolled in the study (Figure 1).

The study was designed as a hybrid trial consisting of both in-person visits and virtual assessments. The study design required participants to complete questionnaires and assess body weight, body fat mass, visceral fat mass, muscle mass, arterial blood pressure and resting heart rate with the aid of a Withings scale (Withings Body Smart (black): 3700546708190; Withings, Issy-Les-Moulineaux, France) at home at weekly intervals, and to attend their local Quest Diagnostics for blood tests and for measuring waist circumference before and at the end of the intervention period. Consent forms describing the study process, instructions, evaluation methods, and bill of rights were provided to participants before inclusion in the study. Medical history was used solely for this research. All documents relevant to the study, which may have included medical history, such as completed consent forms (ICFs), were hosted on a secure online portal provided by Citruslabs (the CRO). The ICFs were completed via HelloSign, secure verification software (also compliant with the Health Insurance Portability and Accountability Act (HIPAA)). All available measures were taken to protect the privacy rights and welfare of participating individuals. These measures included, but were not limited to, secure data storage (through password and encryption), data management access for authorized personnel only and anonymization of individual subject records. Access to the information was restricted to only those who had a need to know for the performance of their job. The patient ID numbers were assigned after consent was obtained, and any link between study data and medical history was prevented. Email addresses and phone numbers were used only for communication with the individuals: a) to schedule onboarding to the secure patient portal, and b) in case individuals had questions. The addresses of individuals were only used by Citruslabs to ship the test products to the participating individuals. No medical history was transferred to the sponsor of the study (SlimBiotics).

Following the consent process, participants attended in-person blood draws and completed the Baseline questionnaire, which included answering validated questionnaires about their gastrointestinal health, eating patterns, stress, and anxiety. The validated questionnaires were:

- Three-Factor Eating Questionnaire (TFEQ) [22];
- Perceived Stress Scale (PSS) [23,24];
- Generalized Anxiety Disorder (GAD-7) [25,26].

In-person visits were required at Baseline and Week 12 to obtain blood draws from participants for biomarker measurement. Virtual assessments were conducted weekly (digital tracking of body weight, muscle mass, fat mass, systolic and diastolic blood pressure, and resting heart rate), and study questionnaires were completed at baseline and at Week 4, Week 8, and Week 12.

### 2.3. Inclusion Criteria

Adult women and men (18 years or older) with a BMI between 25 and 32 (average BMI of subjects should not exceed 30) were included if they were considered healthy, meaning that they did not suffer from an uncontrolled chronic disease. Furthermore, they had to own a sleep-tracking device.

### 2.4. Exclusion Criteria

- Any pre-existing chronic conditions that would prevent participants from adhering to the protocol, including oncological and psychiatric disorders.
- Anyone with a history of severe allergic manifestations.
- Women who are pregnant, breastfeeding or attempting to become pregnant.
- Unwilling to follow the study protocol.
- Subjects currently enrolled in another clinical study.
- Subjects who finished another clinical study within the last 4 weeks before inclusion.
- Hypersensitivity, allergy or intolerance to any compound of the test products.
- Condition after implantation of a cardiac pacemaker or other active implants.
- Sulfonylurea treatment.
- Any disease or condition that might significantly compromise the hepatic (ascites), hematopoietic, renal, endocrine, pulmonary, central nervous, cardiovascular, immunological, dermatological, gastrointestinal or any other body system, with the exception of overweight as defined by the inclusion criteria.
- History or presence of liver failure as defined by Quick index < 70%.
- History of ascites.
- History of hepatitis B, hepatitis C, or HIV.
- Regular medical treatment, including over-the-counter products, which may have an impact on the study aims (e.g., probiotic-containing supplements, laxatives, steroids, etc.).
- Subjects who are scheduled to undergo any diagnostic intervention or hospitalization, which may cause protocol deviations.
- Simultaneous study participation by members of the same household.
- Any diet intended to lose body weight.
- Eating disorders or a vegan diet.
- Anorexic drugs.
- Current drug abuse or alcoholism.

### 2.5. Test Products

#### 2.5.1. Placebo

The main component of the placebo was microcrystalline cellulose (MC). MC is refined wood pulp, a connective agent added to prescription drugs, over-the-counter medications, and dietary supplements. It is a white, free-flowing powder. Chemically, it is an inert substance, is not degraded during digestion and has no appreciable absorption. In large quantities above the range used in the test products, it provides dietary bulk and may have a laxative effect. The active ingredient (postbiotic) used in this study was provided by Centro Sperimentale del Latte S.r.l. (CSL), Strada per Merlino, 326839 Zelo Buon Persico, Italy. The final test products were produced by MeriCal, 447 W. Freedom Ave, Orange, CA 92865, USA. The placebo contained the same components as the postbiotic test product besides the active ingredient (Table 1a). All study test products were similar in weight and appearance.

#### 2.5.2. Postbiotic

The postbiotic test product contained inanimate *L. fermentum* strain K8-Lb1 (DSM 22832) [12,13].

Strain culture, heat treatment and microbial analyses were performed by CSL, certified for production of bacterial cultures for food and farming/livestock, pharmaceutical and nutraceutical sectors. The company CSL has been assessed and complies with the requirements of FSSC 22000 (certification scheme for food safety systems).

The species *L. fermentum* is included in EFSA’s Quality Presumption of Safety (QPS) list. The strains were shown to be sensitive to antibiotics, following EFSA guidelines and ISO/IDF standards [27,28]. The content of the postbiotic cells per capsule was 10^10^ (Table 1). Daily intake was intended to be 1 capsule equivalent to 1 × 10^10^ postbiotic cells.

##### Mode of Consumption

Each study participant was instructed to consume one capsule daily with a meal for 12 weeks, without prescribing which meal during the day it should be.

### 2.6. Statistics

#### 2.6.1. Conduct

The statistical analysis was performed by the Clinical Research Center Kiel.

#### 2.6.2. Determination of Sample Size

The sample size of *n* = 30 per group was used for budgetary reasons in this pilot trial. Based on the results of a DB-RCT with the pertinent viable strains [21] and assuming a 30% higher effect size for the postbiotic strain K8-Lb1 versus the probiotic product (based on data in *C. elegans*), while taking the standard deviation of the Placebo group into account (best-case scenario), a sample size of *n* = 47 per group was calculated for BFM, aiming for a probability of 80% to attain a significant result (power = 0.8).

#### 2.6.3. Definition of Sets to Be Analyzed

##### Full Analysis Set (FAS)

Compliance with the ITT principle would necessitate complete follow-up of all randomized subjects for study outcomes. As this cannot be achieved in most studies, a full analysis set (FAS) was planned to be analyzed to provide evidence for an effect. This is as complete as possible and as close as possible to the ITT-set (FAS), including all randomized test persons. Elimination of individuals is considered to be justified according to the ICH E9 Guideline in the following cases:

- Violation of an essential, before randomization objectively measurable inclusion criterion;
- Not taking a single dose of the test substance (without knowledge of the assigned test group);
- Lack of any data for the judgment of effectiveness after randomization.

##### Intention-To-Treat (ITT) Set

Evaluation in the ITT and PP population served as sensitivity analyses with regard to the primary parameter (BFM). ITT was defined as all individuals randomized and having taken at least one dose of the test products (at V1). Subjects were evaluated in the planned treatment regimen rather than the actual treatment given.

##### Per-Protocol (PP) Set

The PP set was defined as all randomized individuals who had no major protocol deviation.

The per-protocol analysis was used for checking the robustness of the product effect.

#### 2.6.4. Statistical Tests

##### Null Hypothesis

In the case of normal distribution, the null hypothesis was: There is no difference between the mean values of the Postbiotic and Placebo (control) groups concerning the parameters to be tested according to the parameter description in the study protocol. If data are not normally distributed, the null hypothesis assumes an equality of the medians of both groups concerning the parameters to be tested.

##### Alternative Hypothesis

In the case of normal distribution, the mean values of the parameters to be tested acc. to the parameter description in the study protocol differ between both groups. If data are not normally distributed, the parameter medians differ between both groups.

##### Testing of Normality

All metric data were tested for normal distribution using the Shapiro–Wilk test. In order to reduce the risk of falsely rejecting normality (Type I error), which would unnecessarily route data to the less powerful non-parametric test, the null hypothesis (data are normally distributed) was tested at a level of 2.5%. If *p* > 0.025, the null hypothesis was confirmed, and the data were considered normally distributed. The test for normality failed if *p* ≤ 0.025. This conservative approach is consistent with recommendations by Razali & Wah (2011) [29] and is common practice when normality testing serves as a decision rule for downstream test selection.

##### Statistical Tests for Comparison of the Groups

A separate statistical analysis plan (SAP) beyond what was defined in the study protocol document was not written prior to study conduct. The strategy for evaluation, including expressing alterations during intervention as a percentage of baseline, the definition of the primary endpoint (body fat mass), the between-group comparison framework, and the sample size rationale, was defined before unblinding.

In fact, all parameter values were normalized to the baseline and tested as a percent from baseline between groups. Tests were performed at a significance level of 5%, and *p* values in tables are highlighted in green. A trend toward a significant difference was defined as a *p* value between 0.05 and 0.1 and highlighted in yellow.

In case of normality, the Welch’s test was used for statistical analysis to compare both groups (no homoscedasticity was assumed). Figures are shown as mean + SD where data were normally distributed and as box plots where they were not. If the Shapiro–Wilk test for normality failed, the Mann–Whitney U test was performed, and figures are presented as box plots with a dashed line representing the mean value.

After reviewing the raw data, several typing errors (zero and negative values) and evidently swapped values of the self-assessed parameters of body fat mass, muscle mass, visceral fat mass, systolic and diastolic blood pressure were found. Evaluation was made after deleting or correcting evident typing errors and after correcting evidently swapped values. Because any dieting during the study was not allowed (exclusion criterion), it was considered that values deviating more than 10% from baseline in body fat mass, visceral fat mass, muscle mass and body weight were not acceptable outliers. Thus, for visceral fat mass, 5 values from week 0 were replaced with values at week 1 in case of a >10% difference between week 0 and week 1 (NOCB—Next Observation Carried Backward), assuming initial operation errors by the participants. In addition, all outliers in weeks 1 to 12 with a >10% deviation from baseline (or the corrected baseline) in fat mass, visceral fat mass, muscle mass and body weight were omitted.

##### Descriptive Statistics

For descriptive statistics, data are expressed as mean, standard deviation (SD), median, 25th and 75th percentiles, and frequency of observation.

#### 2.6.5. Approach to Treatment of Missing Values

The missing values of the primary parameter Body Fat Mass were subjected to analysis in order to assess the robustness of the primary analysis. The distribution of missing values in both groups was compared by means of a Chi-square test at a 5% significance level.

#### 2.6.6. Interim Analysis

Interim analysis was neither planned nor performed.

#### 2.6.7. Software

SigmaPlot 14.0 (Systat Software, Inc., San Jose, CA, USA) was the main statistical programming software for this study.

## 3. Results

### 3.1. Key Data of Study Conduct

**Figure 2:**
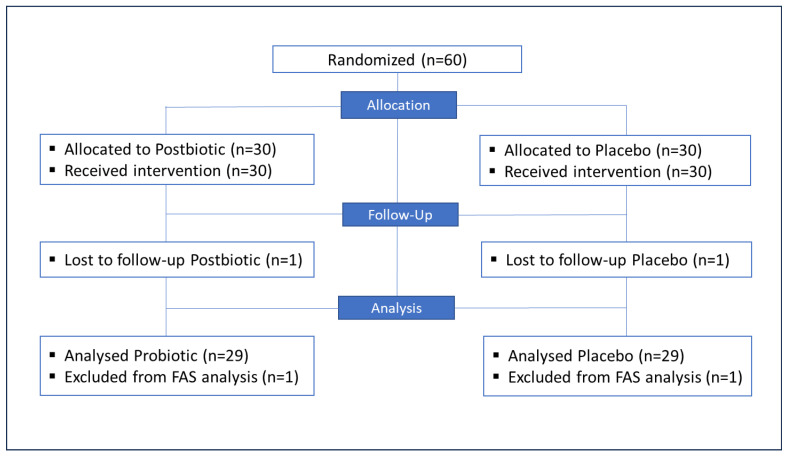
Flow diagram of the study progress through the different phases (according to CONSORT).

As intended, *n* = 60 subjects were randomized and allocated to the Postbiotic and Placebo groups, with 30 subjects each (Figure 2).

### 3.2. Study Populations

ITT Population

The ITT population in this study consisted of all 60 randomized study participants (25 men and 35 women), with 30 assigned to the Postbiotic group and 30 to the Placebo (control) group.

FAS Population

After the baseline visit, *n* = 2 participants dropped out—one from each group. They had no further measurements of any parameter, so no information was available to calculate the parameter alterations during the study. In this case, due to a lack of any data for the assessment of effectiveness after randomization, these subjects were excluded from the FAS.

Therefore, the FAS consisted of *n* = 58 subjects (29 for each group).

The primary analysis was performed based on this data set.

PP Population

Another three subjects—one from the Postbiotic and two from the Placebo group— did not attend the endline visit. The alterations in waist circumference and in all blood parameters could not be determined for these subjects. Due to this protocol violation, they were excluded from the PP population. Therefore, the PP population consisted of 55 participants—28 from the Postbiotic group and 27 from the Placebo group.

### 3.3. Missing Values

The parameters of body composition, body weight, blood pressure and heart rate were assessed weekly from baseline (week 0) to endline (week 12). A few measurements were missing. Additionally, values that were evidently not correct, e.g., 0 or (−0.1) for visceral fat mass, 20 mmHg for diastolic BP or 771 min^−1^ for heart rate, were deleted and categorized as missing values. Thus, 23 values out of 5278 (0.44%) from these parameters were missing in the FAS.

Blood values and waist circumference were determined at Baseline and Endline. The number of individuals (3; 5.17% from FAS) who did not appear at the endline visit did not differ significantly between the groups in the FAS*:* Chi^2^(2, *n* = 58) = 0.351, *p* = 0.554 (Chi^2^ is categorized as inaccurate because of the very small count of missing values). Overall, all missing values from all parameters in the study amounted to 1.42%. Hence, the missing values could be neglected and were not replaced with imputed values in the primary analysis. The statistical evaluation was performed based on the available case analysis. Imputations were only made for the primary parameter BFM in the ITT population.

### 3.4. Participant Demographic and Baseline Characteristics

The ITT population consisted of 41.67% male and 58.33% female subjects at baseline. The mean age was 44.27 ± 9.36 years (Table 2). The distribution of sex and race of the total ITT population in the Postbiotic group and the Placebo group are shown in Table 2.

Body weight was 84.60 ± 10.42 kg in the Postbiotic group and 86.72 ± 16.10 in the Placebo group. There was no significant difference in body weight, body fat mass, visceral fat mass, muscle mass and vital parameters between the groups at baseline (Table 3).

### 3.5. Impact of the Postbiotic on Waist Circumference, Body Weight, Body Composition and Vital Signs

Results are presented in Table 4.

Starting from baseline values of 84.60 ± 10.42 kg in the Postbiotic and 86.72 ± 16.10 in the Placebo group, *body weight* decreased by 1.79% (−1.18 ± 3.43 kg) in the Postbiotic group and by 0.12% (−0.12 ± 2.39 kg) in the Placebo group. Outliers greater than 10% of baseline were deleted (outliers of up to 58% of baseline were found). The difference between groups was significant (*p* = 0.047). Results are shown in Table 4. Without deleting outliers, *p* = 0.198 was found in week 12.

*Body fat mass* (primary parameter) showed a 1.85% decrease in the Postbiotic group and a 0.41% increase in the Placebo group, and the difference between these changes was statistically significant (*p* = 0.016; Table 4). Without deleting outliers, the groups also differed significantly, with *p* = 0.046 in week 12.

The significant difference in body fat mass was also seen in the ITT population with *p* = 0.011, and in the PP population with *p* = 0.027 (Table 5).

*Visceral fat mass* tended (*p* = 0.053) to decrease in the Postbiotic group compared to the Placebo group (Table 4). Outliers > 10% of baseline were deleted (outliers of up to 985% of baseline were found). Without deleting outliers, *p* = 0.096 was found in week 12.

*Muscle mass* tended (*p* = 0.062) to be increased in the Postbiotic group compared to the Placebo group (Table 4). Outliers greater than 10% deviation from baseline were found during the study but not in week 12.

*Systolic blood pressure* showed no significant difference between Postbiotic and Placebo groups during the 12-week intervention (Table 4). *Diastolic blood pressure* showed a trend to decrease in the Placebo group in week 12, but this result is considered an incidental finding, since it is not in line with other parameters associated with overweight individuals.

No significant difference in *heart rate* was found (Table 4).

### 3.6. Impact of the Postbiotic on the Blood Biomarkers

#### 3.6.1. Liver Function Parameters

*ALT, AST* and *GGT* tended to be decreased in the Postbiotic group compared to the Placebo group during intervention (Table 6).

#### 3.6.2. Inflammation Biomarker

Highly sensitive *CRP,* as a biomarker for inflammation, showed a nominal but not significant decrease in the Postbiotic group compared to the Placebo group (Table 7).

#### 3.6.3. Metabolic Biomarkers

The reduction in HbA1c was close to a trend in the Postbiotic group compared to the Placebo group (*p* = 0.1486, Table 8).

The *estimated average glucose* (eAG) was calculated as *eAG* [mg/dL] = 28.7 × HbA1c − 46.7 and rounded as usual to a whole number. The eAG [mg/dL] was converted into [mmol/L] and rounded as usual to a number with one decimal point.

In the subpopulation with normal fasting glucose levels (<100 mg/dL), where measurements were taken into account before and after intervention, HbA1c showed a trend toward significance (*p* = 0.058) and eAG [mmol/L] differed significantly (*p* = 0.046) between the groups (Table 9). In addition, two parameters were examined in both strata: HOMA (Homeostasis Model Assessment) and QUICKI (Quantitative Insulin Sensitivity Check Index).

#### 3.6.4. Serum Lipids

*Serum lipids* did not significantly differ between the Postbiotic and Placebo groups (Table 10).

### 3.7. Impact of the Postbiotic on Validated Questionnaire Scores for Stress, Anxiety and Eating Behavior

As part of the overall questionnaire, participants were asked to complete three validated questionnaires, including the *Perceived Stress Scale*, *Generalized Anxiety Disorder-*7, and the *Three Factor Eating Questionnaire*, which allowed for the analysis of their stress and anxiety levels, as well as their relationship with food. The answers were transformed using a textual Likert scale, and the sum of each participant’s answers was calculated to find an overall score for each validated questionnaire. For each questionnaire, **lower** scores represented **better** outcomes, **higher** scores **worse** outcomes.

The alterations during intervention did not significantly differ between the groups (Table 11).

### 3.8. Impact of the Postbiotic on Subject-Specific Parameters

Participants were asked to answer individual questions at the end of Week 4, Week 8, and Week 12. These questions measured *control over body weight*, *eating habits*, *physical exercise*, several *sleep parameters*, and overall *mood* and the frequency and intensity of *gastrointestinal symptoms*.

For the eight subject-specific questions, a **higher** score indicates a **better** outcome. For the seven symptom-related questions, a **lower** score indicates a **better** outcome.

At endline assessment, the Postbiotic group tended (*p* = 0.066) to feel in more control of their body weight (Table 12).

At Week 12, for the frequency/intensity of poor concentration/focus, there was a 12.18% decrease in the Postbiotic group and a 22.99% increase in the Placebo group—the difference between these changes was statistically significant (*p* = 0.014; Table 11). This suggests an improvement in the Postbiotic group compared to the Placebo group.

The feeling of nausea tended to be improved in the Postbiotic group compared to the Placebo group (*p* = 0.055).

## 4. Discussion

This study investigated the efficacy of the test product in improving various health-related parameters over 12 weeks. The investigation covered a comprehensive array of measures, including body weight, waist circumference, body composition, vital signs, blood biomarkers, validated questionnaires assessing stress, anxiety, and eating behavior, and specific health-related questions targeting body weight control and related aspects of quality of life, as well as gastrointestinal symptoms, fatigue and the ability to concentrate.

One may argue that the study has some limitations: 1. The rather low sample size of *n* = 30 per group. Even in a best-case estimation, *n* = 47 per group was calculated when a) BFM data of the controlled clinical trial were used in which the effect of three probiotic *L. fermentum* strains was investigated [21], one of which was the strain K8-Lb1, and b) findings in an obesity model of *C. elegans* were considered, which showed about 30% higher effect size of the strain K8-Lb1 compared to the mixture of the three strains used in the clinical trial. 2. The self-assessment of anthropometric and body composition data and a posteriori corrections. In fact, some self-assessment data were obviously not correct, as outlined in paragraph 2.6.4. In an a posteriori approach, such evident errors were eliminated by clearly defined algorithms based on plausibility rationales. 3. Lack of dietary assessment and physical activity monitoring. Although the study participants were asked to maintain an ad libitum diet and not to change physical activity, we cannot exclude that individuals changed any eating habits, since we did not perform a 24 h recall, a food frequency questionnaire or evaluate physical activity. Addressing issues of weight management may influence the participants’ eating and lifestyle habits, even if an ad libitum diet was demanded. This kind of momentum, however, had a potential effect on both groups: the Postbiotic group and the Placebo group. The lack of reduction in body fat, body weight and related parameters in the Placebo group indicates that such effects had no major impact in this study.

The drop-out rate was low, at 3.3% for the objective data and evenly distributed between the Postbiotic and Placebo groups. This makes a bias based on drop-out rate unlikely. The drop-out rate was not exceptionally low compared with other studies in which a supplement without side effects was tested and no lifestyle changes were demanded. In the trial in which the probiotic consisted of three strains, including the one tested here as a postbiotic, the drop-out rate was even lower (2.22%) [21].

After the 12-week intervention, body fat mass (primary parameter) was significantly reduced by 2.26% in the Postbiotic group compared to the Placebo group. Since the alteration also differed significantly between the groups when outliers were not deleted, and since the alteration differed significantly as well in the ITT and PP populations, this finding can be regarded as robust according to ICH E8. The significant reduction in BFM was more than expected in this exploratory study with data from only *n* = 58 individuals.

In line with the loss in body fat mass, body weight and waist circumference were significantly reduced, and visceral fat tended to be reduced (*p* = 0.053). Again, in line with this, individuals of the Postbiotic group showed a trend (*p* = 0.066) towards feeling more in control of their body weight, as indicated by the questionnaire on subject-specific parameters. The difference in weight loss (1.67%) was achieved without any dieting. It was certainly less pronounced than that found with the most effective pharmaceuticals, such as GLP-1 receptor agonists [30]. In contrast to what is seen during weight loss induced by GLP-1 agonists [31], muscle mass tended (*p* = 0.062) to be increased, despite the decrease in body weight and body fat. Further side effects, seen with GLP-1 receptor agonists, such as nausea, vomiting, constipation, and diarrhea, were not observed. We even found a trend for improvement in nausea. For assessment of less frequent but severe side effects reported for GLP-1 receptor agonists, like gastroparesis, gallbladder and biliary disorders, cancer and neovascular age-related macular degeneration [31,32], the sample size of this study was not sufficient.

ALT (*p* = 0.098), AST (*p* = 0.072) and GGT (*p* = 0.086) tended to be reduced, which may indicate a reduction in liver steatosis. Currently, these liver enzymes are the most commonly used serum markers of liver steatosis [33]. Their sensitivity and specificity, however, are limited in diagnosis and in grading of liver steatosis, for which the gold standard is still liver biopsy. In recent years, several non-invasive indices were suggested that show higher predictive value than these enzymes alone. These indices combined liver enzyme values with signs of the metabolic syndrome, such as BMI, waist circumference, HDL-C and insulin resistance [33]. In fact, the trend for a reduction in liver enzymes found in this study was in line with the significant reduction in body fat mass, weight and waist circumference, with which liver steatosis is associated [34]. This makes the link between the trend for a reduction in these enzymes and a reduction in liver steatosis more likely.

In line with the reduction in body fat mass, weight, and waist circumference, HbA1c was reduced (*p* = 0.149) in the Postbiotic group compared to the Placebo group, and the alteration of eAG differed significantly between the groups in individuals with normal fasting glucose levels (<100 mg/dL).

The ability to concentrate, which can be impaired in overweight individuals [35], significantly improved in the Postbiotic group compared to the Placebo group.

The results of this pilot study with a rather small sample size need confirmation by a pivotal trial with prior sample size estimation based on the results of this pilot trial.

The results of this study are in line with animal trials and other human studies showing an effect of postbiotics on overweight and traits of the metabolic syndrome [11,20,36,37]. The most recent meta-analysis provided evidence from 25 randomized clinical trials in humans for an effect of postbiotics on waist circumference, but no significant reduction in body weight and BMI, fasting blood glucose (FBG), insulin resistance (HOMA-IR) or HbA1c [37]. The postbiotic *L. fermentum* K8-Lb1 showed effects on body weight, waist circumference and body fat mass and a reduction in HbA1c and eAG in individuals with normal glucose levels.

This clinical trial was not dedicated to the clarification of the mechanisms involved in exerting the effects on body fat, body weight and related parameters. We also did not assess the effect of the postbiotic strain K8-Lb1 on gut microbiota. There are, however, mechanisms of postbiotics that are independent of a change in intestinal microbiota and of the active metabolism of the administered strain. As outlined in the introduction, proteoglycans at the surface of bacteria were shown to act as ligands for receptors, like toll-like receptors (TLRs) [14] and peptidoglycan recognition proteins (PGlyRPs) [15,16], which can mediate anti-inflammatory properties. Low-grade inflammation and LPS were shown to result in dysregulation of anorexigenic and orexigenic signaling [9]. Furthermore, fragments of proteoglycans, so-called muramyl dipeptides, were found to stimulate GLP-1 [17]. *L. fermentum* strains contain the chaperone ClpL, which seems to operate through mechanisms similar to ClpB [18], which mimics α-MSH, a molecule involved in controlling appetite [19,20].

## 5. Conclusions

This 12-week study explored the effects of a postbiotic formulation on weight management and metabolic health.

After a 12-week intervention, body weight, waist circumference, body fat mass and the ability to concentrate were significantly improved in the Postbiotic group compared to the Placebo group. In line with this, visceral fat, ALT, AST and GGT showed a trend towards reduction, and a reduction in HbA1c and eAG was observed in individuals with normal glucose levels before and after intervention. The results of this pilot trial need confirmation by a pivotal trial.

## Figures and Tables

**Table 1:** (**a**): Composition of the test products. (**b**): Microbiological analysis of the postbiotic product.

(a)		
Ingredients Per Capsule	Placebo	Postbiotic
	mg	mg
Heat-killed *L. fermentum* K8-Lb1		34.0 *
Microcrystalline cellulose 112	227.95	193.95
Magnesium stearate VEG. FG	2.35	2.35
Sylysia 350FCP (SILICA/SIO_2_)	4.7	4.7
Total	235.00	235.00
(**b**)		
**Specifications of the Postbiotic**	**Acceptance Range**	**Results**
Inanimate cells per gram *	>3.0 × 10^11^	3.4 × 10^11^
Viable cells (CFU/g)	<5 × 10^3^	2.8 × 10^3^
*Enterobacteriaceae* (CFU/g)	<10	<10
Yeast and Mould (CFU/g)	<10	<10
Non-lactic acid bacteria (CFU/g)	<5000	<5000
*Bacillus cereus* (CFU/g)	<500	<500
*Escherichia coli* (CFU/g)	n.d.	n.d.
Coagulase positive Staphylococci (incl. *Staphylococcus aureus*) (CFU/g)	n.d.	n.d.
*Salmonella* spp. (CFU/25 g)	n.d.	n.d.
*Listeria monocytogenes* (CFU/25 g)	n.d.	n.d.

* 34 mg contained 10^10^ inanimate cells. The cell count was assessed microscopically using a Thoma chamber. (Taichung City, Tai Wan) The analyses were performed by Centro Sperimentale del Latte S.r.l. (CSL).

**Table 2:** Participant demographic information. Post = Postbiotic, Pla = Placebo.

Parameter		Group	Size	%	Total Size	Total %
Sex	Male	Post	12	40.00	25	41.67
		Pla	13	43.33		
	Female	Post	18	60.00	35	58.33
		Pla	17	56.67		
Race	White	Post	19	63.33	34	56.67
		Pla	15	50.00		
	Hispanic or Latino	Post	5	16.67	8	13.33
		Pla	3	10.00		
	Asian	Post	2	6.67	4	6.67
		Pla	2	6.67		
	Preferred not to answer	Post	4	13.33	14	23.33
		Pla	10	33.33		
**Parameter**		**Group**	**Mean**	**SD**	**Total Mean**	**Total SD**
Age	------	Post	43.03	10.36	44.27	9.36
		Pla	45.50	8.24		

**Table 3:** Body composition, body weight, and vital signs at Baseline.

Parameter	Group	Mean	SD	Median	25%	75%	p Welch‘s Test
Body Weight [kg]	Post	84.60	10.42	83.45	76.44	91.66	0.488
	Pla	86.65	16.1	87.71	73.31	94.31	
Body Fat Mass [%]	Post	33.41	5.08	32.90	28.70	38.35	0.228
	Pla	31.45	7.01	32.30	25.85	37.25	
Visceral Fat Mass [%]	Post	3.56	1.16	3.50	2.63	4.43	0.917
	Pla	3.52	1.23	3.40	2.50	4.20	
Muscle Mass [%]	Post	62.81	4.52	62.70	58.50	67.45	0.141
	Pla	65.08	6.82	64.30	59.00	70.45	
Systolic Blood Pressure [mmHg]	Post	121.97	11.82	125.0	116.0	130.0	0.848
	Pla	123.90	12.68	122.0	116.50	130.5	
Diastolic Blood Pressure [mmHg]	Post	78.76	7.31	82.00	75.50	88.00	0.315
	Pla	82.07	8.49	79.00	76.00	84.00	
Heart Rate [min^−1^]	Post	70.93	9.97	71.00	63.75	76.75	0.229
	Pla	74.72	13.30	74.00	64.00	83.50	

**Table 4:** Alteration of waist circumference, body weight, body composition and vital signs during the intervention. Outliers > 10% from baseline in body weight, body fat mass, visceral fat mass and muscle mass were omitted. Significant *p* values are highlighted in green; trends toward significance (*p* values between 0.05 and 0.1) are highlighted in yellow.

Parameter	Group	Size	Baseline		Endline/Baseline [%]					p	Test
			Mean	SD	Mean	SD	Median	25%	75%		
Waist Circumference [cm]	Post	27	97.37	9.21	96.48	9.55	97.14	92.86	100.00	0.034	Mann–Whitney
	Pla	27	94.45	11.66	101.24	9.69	100.00	97.22	105.56		
Body Weight {kg]	Post	28	84.60	10.42	98.21	3.31	98.56	95.37	100.65	0.047	Welch
	Pla	29	86.72	16.10	99.88	2.85	100.05	98.05	101.51		
Body Fat Mass [%]	Post	27	33.41	5.08	98.15	3.32	98.25	96.64	100.53	0.016	Welch
	Pla	27	31.45	7.01	100.41	3.39	100.79	98.11	102.77		
Visceral Fat Mass [%]	Post	25	3.44	1.23	98.59	3.63	97.87	96.08	101.19	0.053	Welch
	Pla	24	3.70	1.21	101.04	4.87	100.00	98.01	105.42		
Muscle Mass [%]	Post	29	62.81	4.52	101.11	2.97	100.87	99.66	102.05	0.062	Mann–Whitney
	Pla	28	65.08	6.82	99.88	2.85	100.05	98.05	101.51		
Systolic Blood Pressure [mmHg]	Post	29	121.97	11.82	99.61	7.77	99.10	93.98	106.22	0.274	Mann–Whitney
	Pla	28	123.90	12.68	98.36	11.98	96.75	88.54	103.39		
Diastolic Blood Pressure [mmHg]	Post	28	78.76	7.31	101.42	11.18	100.00	96.15	105.80	0.079	Mann–Whitney
	Pla	28	82.07	8.49	96.50	11.99	95.79	87.79	102.65		
Heart Rate [min^−1^]	Post	29	74.28	13.20	102.31	13.63	101.67	96.68	106.88	0.684	Welch
	Pla	27	71.39	10.27	103.76	12.83	103.03	95.59	112.70		

*Waist circumference* decreased by 3.52% in the Postbiotic group and increased by 1.24% in the Placebo group during the intervention. The difference between the groups was significant (*p* = 0.034) (Table 4) without deleting outliers; *p* = 0.198 was found in week 12.

**Table 5:** BFM in ITT (median of each group imputed in case of missing values) and PP.

Parameter	Group	Size	Baseline		Endline/Baseline [%]					p	Test
			Mean	SD	Mean	SD	Median	25%	75%		
Body Fat Mass [%] **ITT**	Post	30	33.40	4.99	98.25	96.61	100.57	57.14	115.38	0.011	Mann–Whitney
	Pla	30	31.48	6.89	100.75	98.84	102.52	74.36	115.79		
Body Fat Mass [%] **PP**	Post	28	33.43	5.17	97.77	3.83	98.02	96.56	100.52	0.027	Mann–Whitney
	Pla	26	31.85	7.01	99.91	4.33	100.75	97.58	102.86		

**Table 6:** Liver function parameters. Trends toward significant differences (*p* = 0.05 to 0.10) were highlighted in yellow).

Parameter	Group	Size	Baseline		Endline/Baseline [%]					p	Test
			Mean	SD	Mean	SD	Median	25%	75%		
AST [U/L]	Post	28	18.43	5.12	100.44	26.98	100.00	88.30	107.02	0.072	Mann–Whitney
	Pla	27	24.37	27.41	108.84	30.91	108.33	94.74	125.00		
ALT [U/L]	Post	28	22.14	11.30	95.74	32.32	98.98	81.95	105.36	0.098	Mann–Whitney
	Pla	27	28.81	39.56	106.68	34.83	107.14	94.74	121.43		
GGT [U/L]	Post	28	21.54	13.18	92.46	31.04	88.75	70.01	107.92	0.086	Mann–Whitney
	Pla	27	32.78	60.92	100.83	25.72	100.00	87.50	112.50		
Alk. Phosphatase [U/L]	Post	28	62.25	20.45	98.26	15.84	97.64	85.40	113.34	0.368	Mann–Whitney
	Pla	27	75.70	49.69	100.11	17.96	101.32	93.33	114.49		
Total Protein [g/dL]	Post	28	7.17	0.33	98.48	5.59	98.56	93.63	103.98	0.364	Welch
	Pla	27	6.99	0.51	99.76	4.73	98.70	97.14	102.99		
Albumin [g/dL]	Post	28	4.43	0.21	99.35	5.63	98.94	95.50	104.01	0.922	Welch
	Pla	27	4.36	0.32	99.48	4.41	100.00	97.62	102.50		
Globulin [g/dL]	Post	28	2.74	0.29	97.38	8.99	96.29	89.38	104.61	0.158	Welch
	Pla	27	2.63	0.40	100.67	8.03 z	100.00	96.15	104.17		
ALB/GLOB	Post	28	1.63	0.19	102.86	9.07	105.88	94.12	110.00	0.110	Welch
	Pla	27	1.70	0.29	99.20	7.63	100.00	93.33	106.25		
Total Bilirubin [mg/dL]	Post	28	0.61	0.25	108.49	45.46	100.00	83.33	129.17	0.677	Mann–Whitney
	Pla	27	0.57	0.29	102.69	33.07	100.00	75.00	133.33		

**Table 7:** Inflammation biomarker: highly sensitive CRP (HS CRP).

Parameter	Group	Size	Baseline		Endline/Baseline [%]					p	Test
			Mean	SD	Mean	SD	Median	25%	75%		
HS CRP [mg/L]	Post	28	2.53	1.32	94.67	68.69	81.08	57.14	115.38	0.462	Mann–Whitney
	Pla	27	3.07	2.74	96.12	42.25	100.00	74.36	115.79		

**Table 8:** Metabolic biomarkers (the estimated average glucose (eAG) was calculated as eAG [mg/dL] = 28.7 × HbA1c − 46.7).

Parameter	Group	Size	Baseline		Endline/Baseline [%]					p	Test
			Mean	SD	Mean	SD	Median	25%	75%		
Glucose [mmol/L]	Post	28	5.22	0.64	101.82	21.71	96.35	91.82	103.81	0.840	Mann–Whitney
	Pla	27	5.77	2.39	97.27	15.31	100.00	88.46	105.75		
Insulin [µU/mL]	Post	28	13.39	9.12	144.71	278.14	85.78	52.51	104.81	0.395	Mann–Whitney
	Pla	27	12.21	9.48	111.86	77.71	94.59	68.80	134.85		
HbA1c [%]	Post	27	5.37	0.62	101.98	4.19	101.85	100.00	103.70	0.149	Mann–Whitney
	Pla	27	5.56	1.10	102.55	4.08	103.51	100.00	105.36		
eAG [mg/dL]	Post	27	107.43	17.85	102.72	5.59	102.70	100.00	105.00	0.476	Welch
	Pla	27	112.81	31.71	103.83	5.82	105.13	100.00	107.89		
eAG [mmol/L]	Post	27	5.95	0.99	102.65	5.56	101.75	100.00	105.00	0.412	Welch
	Pla	27	6.25	1.76	103.93	5.84	104.62	100.00	108.00		

**Table 9:** Metabolic biomarkers—*strata* defined depending on measured fasting blood glucose (BG): stratum *normal BG* (BG < 100 mg/dL); stratum impaired fasting glucose + type 2 diabetes (BG ≥ 100 mg/dL).

Parameter	Stratum	Group	Size	Baseline		Endline/Baseline [%]					p	Test
				Mean	SD	Mean	SD	Median	25%	75%		
Glucose [mg/dL]	**BG < 100 mg/dL** **(normal BG)**	Post	22	89.73	8.14	98.05	8.94	95.97	91.64	103.94	0.811	Welch
		Pla	23	91.61	12.21	98.85	12.96	100.00	88.46	106.45		
Insulin [mU/L]		Post	22	12.16	8.45	158.65	313.30	85.78	52.16	104.17	0.281	Mann–Whitney
		Pla	23	11.63	9.87	118.27	82.53	102.53	69.33	135.63		
HbA1c [%]		Post	21	5.19	0.28	101.03	3.05	101.82	100.00	102.04	0.058	Welch
		Pla	23	5.28	0.38	102.88	3.26	103.51	100.00	105.36		
eAG [mg/dL]		Post	21	102.09	7.95	101.53	4.41	102.70	100.00	103.21	0.059	Welch
		Pla	23	104.78	11.02	104.25	4.84	105.13	100.00	107.90		
eAG [mmol/L]		Post	21	5.65	0.45	101.55	4.36	101.61	100.00	103.64	0.046	Welch
		Pla	23	5.80	0.62	104.38	4.77	104.62	100.00	108.00		
HOMA_IR		Post	22	2.76	2.05	171.22	379.31	78.62	48.42	104.35	0.238	Mann–Whitney
		Pla	23	2.86	3.32	123.32	103.94	97.86	60.93	143.48		
QUICKI		Post	22	0.34	0.04	102.31	10.82	103.79	99.35	110.99	0.300	Welch
		Pla	23	0.34	0.04	101.53	12.32	100.34	94.95	108.10		
Glucose [mg/dL]	**BG ≥ 100 mg/dL** **(IFG + DM2)**	Post	6	110.0	6.75	115.65	43.78	100.08	93.67	128.90	0.914	Mann–Whitney
		Pla	4	175.0	84.08	88.23	25.91	99.22	61.80	103.68		
Insulin [mU/L]		Post	6	17.92	10.83	93.62	38.85	85.27	59.79	132.30	0.330	Welch
		Pla	4	15.55	6.85	75.02	16.13	74.83	59.51	90.73		
HbA1c [%]		Post	6	6.05	1.03	105.30	6.08	102.81	101.21	110.31	0.358	Welch
		Pla	4	7.18	2.33	100.65	7.81	102.79	92.59	106.57		
eAG [mg/dL]		Post	6	127.00	29.48	106.85	7.62	103.92	101.46	113.39	0.420	Welch
		Pla	4	159.00	66.99	101.45	10.62	103.91	90.52	109.92		
eAG [mmol/]		Post	6	7.05	1.61	106.49	7.87	103.38	101.03	112.70	0.452	Welch
		Pla	4	8.83	3.71	101.35	10.86	103.51	90.28	110.26		
HOMA_IR		Post	6	4.89	2.95	114.73	79.07	84.37	57.79	181.08	0.281	Mann–Whitney
		Pla	4	6.82	5.43	68.75	29.85	74.20	38.18	93.87		
QUICKI		Post	6	0.31	0.02	100.78	8.38	102.31	92.47	107.79	0.822	Welch
		Pla	4	0.30	0.02	107.19	9.06	104.10	100.81	116.66		

**Table 10:** Lipid profile.

Parameter	Group	Size	Baseline		Endline/Baseline [%]					p	Test
			Mean	SD	Mean	SD	Median	25%	75%		
Total Cholesterol [mg/dL]	Post	28	188.25	35.68	100.56	14.01	99.18	89.79	108.74	0.792	Welch
	Pla	27	195.93	26.53	99.68	10.19	100.00	92.90	104.25		
HDL Cholesterol [mg/dL]	Post	28	52.82	15.32	104.02	20.52	100.00	91.17	108.12	0.794	Mann–Whitney
	Pla	27	55.89	12.15	102.00	14.63	102.00	95.74	107.32		
Triglycerides [mg/dL]	Post	28	114.46	51.65	99.76	27.52	98.88	75.61	117.17	0.768	Mann–Whitney
	Pla	27	121.56	65.96	101.99	36.60	94.89	73.60	121.36		
LDL Cholesterol [mg/dL]	Post	28	113.79	29.22	100.72	20.02	96.56	86.95	111.91	0.775	Welch
	Pla	27	117.44	22.53	99.36	14.65	101.82	88.79	106.82		
CHOL/HDL-C	Post	28	3.75	0.98	99.04	17.33	96.93	87.93	106.45	0.983	Welch
	Pla	27	3.66	0.86	99.13	14.12	97.87	94.29	105.56		
NON-HDL-C [mg/dL]	Post	28	135.43	30.32	99.95	18.31	96.39	90.22	111.84	0.813	Mann–Whitney
	Pla	27	140.04	26.04	98.93	13.09	100.53	90.43	105.43		

**Table 11:** PSS, GAD-7, and TFEQ.

Normalized Value	Parameter	Group	Size	Mean	SD	Median	25%	75%	p	Test
**Week 12/baseline [%]**	PSS	Post	29	97.88	26.35	94.74	79.36	108.35	0.809	Mann–Whitney
		Pla	29	95.91	27.93	90.91	77.82	112.91		
	GAD-7	Post	29	107.21	43.95	92.31	80.91	120.54	0.708	Mann–Whitney
		Pla	29	107.96	39.13	100.00	75.25	133.33		
	TFEQ	Post	29	95.60	17.27	90.74	86.69	107.47	0.539	Welch
		Pla	29	93.17	12.17	93.33	84.24	101.11		

**Table 12:** Subject-specific parameters.

Norm. Value	Question	Group	Size	Mean	SD	Median	25%	75%	p	Test
**NecessaryWeek 12/Baseline [%]**	Q1: Over the 4 weeks. I felt in control of my body weight	Post	29	155.17	95.96	133.33	100.00	200.00	0.066	Mann–Whitney
		Pla	29	115.81	45.55	100.00	100.00	133.33		
	Q2: Over the past 4 weeks. I felt in control of my eating habits and my healthy diet.	Post	29	118.39	49.87	100.00	100.00	133.33	0.713	Mann–Whitney
		Pla	29	128.33	60.54	100.00	90.00	183.33		
	Q3: How often do you implement physical exercise over the past 4 weeks?	Post	29	107.47	34.60	100.00	100.00	100.00	0.252	Mann–Whitney
		Pla	29	95.00	23.59	100.00	77.50	100.00		
	Q4: How would you rate your sleep quality in terms of deep. restful sleep over the past week?	Post	29	99.43	37.86	100.00	66.67	133.33	0.678	Mann–Whitney
		Pla	29	102.87	32.68	100.00	87.50	133.33		
	Q5: How many hours of sleep did you get on average each night over the past week?	Post	29	99.71	17.54	100.00	92.15	111.28	0.382	Welch
		Pla	27	104.32	22.22	100.00	89.60	116.67		
	Q6: What was the sleep quality score you achieved over the past week?	Post	29	101.03	31.70	101.15	89.98	109.93	0.858	Mann–Whitney
		Pla	29	105.85	30.65	100.00	89.74	110.18		
	Q7: How would you rate your overall mood over the past 4 weeks?	Post	29	97.30	17.94	100.00	100.00	100.00	0.341	Mann–Whitney
		Pla	29	103.62	27.52	100.00	77.50	133.33		
	Q8: I felt calm and relaxed over the past 4 weeks.	Post	29	111.49	31.84	100.00	100.00	133.33	0.316	Mann–Whitney
		Pla	29	111.95	43.36	100.00	100.00	125.00		
**Rate the frequency and intensity of the following symptoms over the past week**										
**Norm. Value**	**Symptom**	**Group**	**Size**	**Mean**	**SD**	**Median**	**25%**	**75%**	**p**	**Comment**
**Week 12/baseline [%]**	Nausea and/or vomiting	Post	29	100.00	32.73	100.00	100.00	100.00	0.055	Mann–Whitney
		Pla	29	118.97	43.12	100.00	100.00	100.00		
	Constipation	Post	29	101.38	41.64	100.00	100.00	100.00	0.243	Mann–Whitney
		Pla	29	121.55	69.99	100.00	100.00	100.00		
	Diarrhea	Post	29	123.85	64.15	100.00	100.00	200.00	0.173	Mann–Whitney
		Pla	29	98.28	24.94	100.00	100.00	100.00		
	Belching and/or flatulence	Post	29	100.23	49.09	100.00	50.00	100.00	0.393	Mann–Whitney
		Pla	29	127.30	93.80	100.00	58.33	200.00		
	Bloating	Post	29	102.53	59.12	100.00	50.00	125.00	0.815	Mann–Whitney
		Pla	29	95.40	45.13	100.00	50.00	100.00		
	Poor concentration/focus	Post	29	87.82	58.24	50.00	50.00	100.00	0.014	Mann–Whitney
		Pla	29	122.99	65.83	100.00	58.33	200.00		
	Fatigue and/or sluggishness	Post	29	116.49	65.96	100.00	55.00	200.00	0.526	Mann–Whitney
		Pla	29	99.14	45.55	100.00	62.50	100.00		

## Data Availability

The datasets produced and analyzed in the present study are not publicly accessible at this time. However, they can be made available by the corresponding author upon receiving a justifiable request.

## Abbreviations

α-MSH	α-Melanocyte-stimulating hormone
ALT	Alanine transaminase
AST	Aspartate transferase
BFM	Body fat mass
BMI	Body mass index
CCK	Cholecystokinin
CFU	Colony-forming units
ClpB	Caseinolytic peptidase B
ClpL	Caseinolytic peptidase L
CRO	Contract research organization
CRP	C-reactive protein
DSM	Deutsche Sammlung von Mikroorganismen (German Collection of Microorganisms and Cell Cultures)
eAG	Estimated average glucose
EFSA	European Food and Safety Agency
FAS	Full analysis set
GAD-7	Generalized Anxiety Disorder questionnaire
GGT	Gamma-glutamyl transferase
GLP-1	Glucagon-like peptide-1
HbA1c	Hemoglobin A1c
HDL	High-density lipoprotein
HIV	Human Immunodeficiency Virus
HOMA	Homeostasis Model Assessment QUICKI (Quantitative Insulin Sensitivity Check Index
ICF	Informed consent form
ICH	International Council for Harmonisation of Technical Requirements for Pharmaceuticals for Human Use
IDF	International Dairy Federation
ITT	intent-to-treat
ISO	International Organization for Standardization
LDL	Low-density lipoprotein
LPS	Lipopolysaccharides
PBMC	Peripheral blood mononuclear cells
PGlyRPs	Peptidoglycan recognition proteins
PP	per-protocol
PSS	Perceived Stress Scale
QUICKI	Quantitative Insulin Sensitivity Check Index
SAP	Statistical analysis plan
SCFAs	Short-chain fatty acids
SD	Standard deviation
TFEQ	Three-Factor Eating Questionnaire
Th1	T-helper cell 1
Th2	T-helper cell 2
V	Visit
